# Acceptance of Simulated Adult Patients With Medicaid Insurance Seeking Care in a Cancer Hospital for a New Cancer Diagnosis

**DOI:** 10.1001/jamanetworkopen.2022.22214

**Published:** 2022-07-15

**Authors:** Victoria A. Marks, Walter R. Hsiang, James Nie, Patrick Demkowicz, Waez Umer, Afash Haleem, Bayan Galal, Irene Pak, Dana Kim, Michelle C. Salazar, Elizabeth R. Berger, Daniel J. Boffa, Michael S. Leapman

**Affiliations:** 1 Department of Urology, Yale School of Medicine, New Haven, Connecticut; 2 University of California San Francisco; 3 The College of New Jersey, Ewing; 4 Yale University, New Haven, Connecticut; 5 Department of Surgery, Yale School of Medicine, New Haven, Connecticut; 6 Yale Cancer Outcomes, Public Policy, and Effectiveness Research Center, New Haven, Connecticut

## Abstract

**Importance:**

Although there have been significant increases in the number of US residents insured through Medicaid, the ability of patients with Medicaid to access cancer care services is less well known.

**Objective:**

To assess facility-level acceptance of Medicaid insurance among patients diagnosed with common cancers.

**Design, Setting, and Participants:**

This national cross-sectional secret shopper study was conducted in 2020 in a random sample of Commission on Cancer–accredited facilities in the United States using a simulated cohort of Medicaid-insured adult patients with colorectal, breast, kidney, and melanoma skin cancer.

**Exposures:**

Telephone call requesting an appointment for a patient with Medicaid with a new cancer diagnosis.

**Main Outcomes and Measures:**

Acceptance of Medicaid insurance for cancer care. Descriptive statistics, χ^2^ tests, and multivariable logistic regression models were used to examine factors associated with Medicaid acceptance for colorectal, breast, kidney, and skin cancer. High access hospitals were defined as those offering care across all 4 cancer types surveyed. Explanatory measures included facility-level factors from the 2016 American Hospital Association Annual Survey and Centers for Medicare & Medicaid Services General Information database.

**Results:**

A nationally representative sample of 334 facilities was created, of which 226 (67.7%) provided high access to patients with Medicaid seeking cancer care. Medicaid acceptance differed by cancer site, with 319 facilities (95.5%) accepting Medicaid insurance for breast cancer care; 302 (90.4%), colorectal; 290 (86.8%), kidney; and 266 (79.6%), skin. Comprehensive community cancer programs (OR, 0.4; 95% CI, 0.2-0.7; *P* = .007) were significantly less likely to provide high access to care for patients with Medicaid. Facilities with nongovernment, nonprofit (vs for-profit: OR, 3.5; 95% CI, 1.1-10.8; *P* = .03) and government (vs for-profit: OR, 6.6; 95% CI, 1.6-27.2; *P* = .01) ownership, integrated salary models (OR, 2.6; 95% CI, 1.5-4.5; *P* = .001), and average (vs above-average: OR, 6.4; 95% CI, 1.4-29.6; *P* = .02) or below-average (vs above-average: OR, 8.4; 95% CI, 1.5-47.5; *P* = .02) effectiveness of care were associated with high access to Medicaid. State Medicaid expansion status was not significantly associated with high access.

**Conclusions and Relevance:**

This study identified access disparities for patients with Medicaid insurance at centers designated for high-quality care. These findings highlight gaps in cancer care for the expanding population of patients receiving Medicaid.

## Key Points

**Question:**

Can individuals insured by Medicaid access cancer care services at high-performing cancer-designated hospitals?

**Findings:**

In this cross-sectional study of 334 facilities recognized for cancer care, 95.5% accepted new patients with Medicaid for breast cancer, 90.4% for colorectal cancer, 86.8% for kidney cancer, and 79.6% for skin cancer (melanoma) care. Medicaid was accepted for all 4 surveyed cancers at 67.7% of facilities.

**Meaning:**

Despite increases in the number of US residents insured through Medicaid, these findings suggest that barriers to accessing cancer care exist at high-quality centers.

## Teaser

This cross-sectional study evaluates facility-level acceptance of Medicaid insurance among patients diagnosed with common cancers.

## Introduction

Following significant expansions associated with the Patient Protection and Affordable Care Act (ACA) of March 2010, Medicaid has become the largest social insurance program and third largest payer in the United States, now providing health insurance to 1 in 5 US residents.^1^ As Medicaid has long been an essential source of coverage for low-income, nonelderly US adults with cancer, implementation of the ACA has also led to increases in insurance rates, screening, and access to treatment.^2,3,4,5,6,7,8^ Coverage has notably increased for historically disadvantaged populations of patients with cancer, including members of racial and ethnic minority groups, those residing in rural areas, and individuals with lower educational levels.^4,9^ Despite improvements attributable to ACA expansion, infrastructural and financial constraints remain obstacles to timely and universal care.

Patients with cancer and Medicaid insurance have faced significant barriers to health care, including reduced access to and delays in care.^10,11,12,13^ Most factors that limit a hospital or physician’s participation in Medicaid have not changed, including low reimbursement, high administrative burden, and limited specialist participation in managed care organization networks.^4,11^ Thus, acquiring health insurance through Medicaid does not guarantee access to the multitude of health services required for contemporary cancer care.^14^ Although there are often clear financial and administrative incentives for health care practices to restrict access for patients with Medicaid, the prevalence of this practice, and the facility-level factors associated with its persistence, remain largely unexplored.

With a growing share of US residents insured through Medicaid, a better understanding of potential barriers to cancer care is needed to ensure that the program’s objectives of improving health care for adults with low income can be fully realized. Therefore, we sought to evaluate access to cancer care for patients with Medicaid insurance at Commission on Cancer (CoC)–accredited hospitals, a designation provided to institutions providing standard-setting, comprehensive cancer care.^15^ Focusing on colorectal, breast, kidney, and skin (melanoma) cancer—common cancers detected at an early stage through screening or incidentally through diagnostic imaging—we conducted a cross-sectional national secret shopper study to characterize access to cancer care at CoC hospitals. We hypothesized that despite increases in the number of Medicaid-insured individuals, access barriers would persist across institutions.

## Methods

### Study Design

We conducted a cross-sectional secret shopper study between March and November 2020. This study design has been shown to effectively evaluate appointment availability and care access using simulated patients to call physician offices and attempt to schedule appointments.^11,16,17,18,19^ Trained investigators separately contacted each specialty department of interest at each facility posing as an individual seeking care for a patient with Medicaid and a new cancer diagnosis. Investigators recorded binary appointment availability (yes or no), acceptance of state basic Medicaid (yes or no), and necessity of referral (yes, no, or depends). In line with prior studies, access for patients with commercial insurance and Medicare was assumed to be near 100%.^11,20^ Our study received nonhuman participant research institutional review board exemption from the Yale School of Medicine. This report follows the Strengthening the Reporting of Observational Studies in Epidemiology (STROBE) reporting guidelines for cross-sectional studies.

The primary study objective was to identify facilities providing high access to cancer care services for patients with Medicaid, defined as appointment availability for all 4 cancer types surveyed (colorectal, breast, kidney, and skin). The secondary objectives were to identify characteristics associated with high-access facilities and differences in Medicaid access for individual cancer sites.

### Facility Identification and Characteristics

We identified facilities using the American College of Surgeon’s Commission on Cancer Hospital Locator.^15^ We excluded hospitals with unique Medicaid policies, specialty hospitals, facilities in Puerto Rico, hospitals not characterized in the 2016 American Hospital Association (AHA) Annual Survey or Centers for Medicare & Medicaid Services (CMS) General Information database, and hospitals that did not treat all investigated cancer types (eFigure in the Supplement). We used a random number generator to construct a representative sample of approximately one-third of CoC-accredited hospitals that met inclusion criteria.

We compiled facility-level characteristics hypothesized to be associated with acceptance of Medicaid from the 2016 AHA Annual Survey and CMS General Information databases.^1,21,22^ The AHA database provided hospital-reported information on services offered, organizational structure, utilization, and finances.^21^ The CMS database was used to provide data on hospital performance.^22^ Using AHA data, we examined patient entry points to care via presence of emergency department or outpatient center and whether the facility was the sole provider within the community. We queried organization accreditations including facility type, teaching hospital status, and medical school affiliation. We evaluated total facility capacity via total facility admissions and Medicaid discharges (ie, discharges where Medicaid managed care plan was the payment source). We investigated facility financial characteristics such as ownership, fee-for-service model, integrated salary model, and accountable care organization (ACO) status. We used CMS data to explore the association between Medicaid acceptance and facility performance through ranked variables, including hospital overall rating, effectiveness of care, and timeliness of care. Finally, we recorded whether facilities were located in a Medicaid expansion state at time of data collection.^23^

### Statistical Analysis

The Pearson χ^2^ test and multivariable logistic regression were used to evaluate associations between facility characteristics and Medicaid access. Variables that approached significance on univariable analysis (*P* < .10) were included in the multivariable model. Continuous variables were grouped into quintiles, and the highest quintile was compared against the lower 4 quintiles. Individual analyses were conducted for each cancer type. Statistical analysis was performed using JMP version 15.0 and Stata/IC version 15.0 (StataCorp). Facility locations and corresponding telehealth availability were mapped using ArcGIS software (Esri) (Figure). State boundaries were extracted from USA States (Generalized) data layer. *P* < .05 was considered statistically significant, and all tests were 2-tailed.

**Figure.**
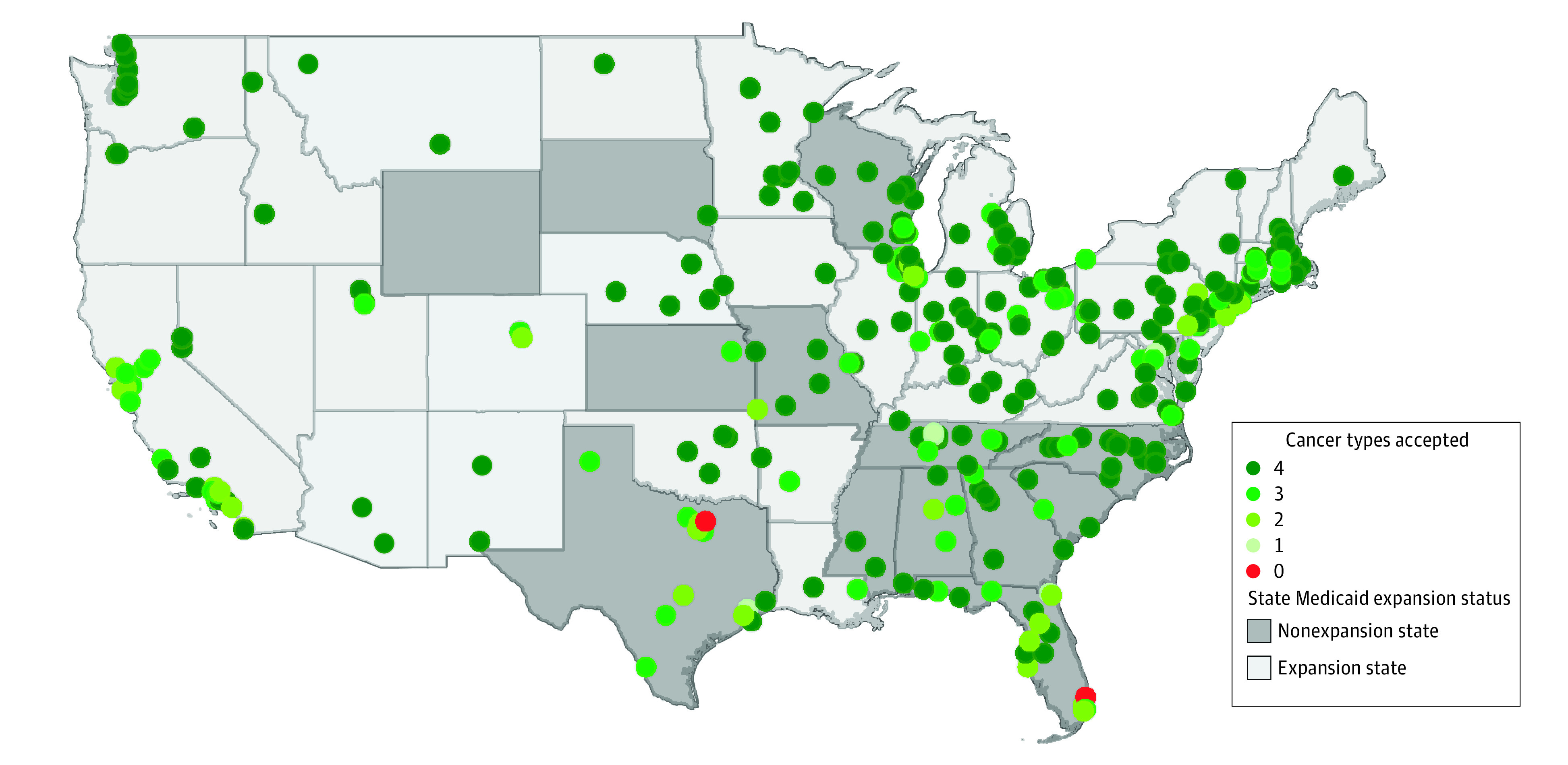
Medicaid Acceptance at Investigated Commission on Cancer–Accredited Facilities Across the United States Four cancer types (colorectal, breast, kidney, and skin [melanoma]) were queried. This map was generated using ArcGIS software by Esri. State boundary data was extracted from States (Generalized) publicly available data set.

## Results

### Facility-Level Acceptance of Medicaid by Cancer Type

We contacted 334 facilities, of which, 226 (67.7%) accepted Medicaid for all 4 cancer types and were characterized as high access (eFigure in the Supplement). Acceptance varied within facilities, with 296 facilities (88.6%) accepting Medicaid for at least 3 cancer types, 324 (97.0%) for at least 2, and 331 (99.1%) for at least 1 cancer type (Table 1). Hospital-level Medicaid acceptance for colorectal, breast, kidney, and skin cancer was 90.4% (302 facilities), 95.5% (319 facilities), 86.8% (290 facilities), and 79.6% (266 facilities), respectively (Table 1). The Figure demonstrates variation in Medicaid acceptance across investigated facilities.

**Table 1.** Medicaid Acceptance by Cancer Type

Medicaid acceptance	CoC facilities, No. (%) (N = 334)
Cancer departments accepting Medicaid within facility	
At least 1 cancer type	331 (99.1)
At least 2 cancer types	324 (97.0)
At least 3 cancer types	296 (88.6)
All 4 cancer types (high-access facilities)	226 (67.7)
Medicaid acceptance by cancer type across facilities	
Colorectal	302 (90.4)
Breast	319 (95.5)
Kidney	290 (86.8)
Skin (melanoma)	266 (79.6)

Abbreviation: CoC, Commission on Cancer.

### Characteristics of Facilities With High Medicaid Acceptance

Table 2 details facility characteristics and distribution of our sample. Several key facility-level characteristics were associated with high access hospitals. In univariable analysis, high-access hospitals were more commonly located within Medicaid expansion states than nonexpansion states (164 of 230 [71.3%] vs 62 of 104 [59.6%]; *P* = .04). National Cancer Institute (NCI)–designated cancer programs were more commonly high-access (26 of 29 [89.7%]), followed by academic comprehensive (38 of 44 [86.4%]), community (56 of 75 [74.7%]), integrated network (25 of 36 [69.4%]), and comprehensive community (81 of 150 [54.0%]) cancer programs (*P* *<* .001). For-profit hospitals (16 of 38 [42.1%]) were less likely to be high access facilities than nongovernment, nonprofit (179 of 257 [69.6%]) and government facilities (31 of 39 [79.5%]) (*P* *<* .001). Major teaching hospitals (59 of 73 [80.8%] vs 167 of 261 [64.0%]; *P* = .007) and hospitals with medical school affiliations (153 of 212 [72.2%] vs 73 of 122 [59.8%]; *P* = .02) were more likely to be high access hospitals than those without accreditations. High access to Medicaid was also associated with integrated salary models (144 of 191 [75.4%] vs 82 of 143 [57.3%]; *P* < .001), ACOs (125 of 170 [73.5%] vs 78 of 126 [61.9%]; *P* = .03), higher total facility admissions (52 of 67 [77.6%] vs 173 of 266 [65.0%]; *P* = .05), and an average (190 of 280 [67.9%]) or below-average (28 of 36 [77.8%]) effectiveness of care ranking compared with an above-average ranking (6 of 15 [40.0%]) *(P* *=* .03).

**Table 2.** Univariable Analysis of Hospital Characteristics Associated With Medicaid Acceptance at Hospitals With vs Without High Access to Medicaid

Hospital characteristics	Facilities, No. (%)			*P* value
	Total CoC facilities, (n = 334)	High-access-to-Medicaid facilities, (n = 226)	Not high-access facilities, (n = 108)	
State expansion status				
Nonexpansion state	104 (31.1)	62 (59.6)	42 (40.4)	.04
Expansion state	230 (68.9)	164 (71.3)	66 (28.7)	
Facility type				
Community	75 (22.5)	56 (74.7)	19 (25.3)	<.001
NCI designated	29 (8.7)	26 (89.7)	3 (10.3)	
Integrated network	36 (10.8)	25 (69.4)	11 (30.6)	
Academic comprehensive	44 (13.2)	38 (86.4)	6 (13.6)	
Comprehensive community	150 (44.9)	81 (54.0)	69 (46.0)	
Ownership				
For-profit	38 (11.4)	16 (42.1)	22 (57.9)	<.001
Nongovernment nonprofit	38 (76.9)	179 (69.6)	78 (30.4)	
Government	257 (11.7)	31 (79.5)	8 (20.5)	
Sole community provider				
No	6 (1.8)	220 (97.3)	108 (100.0)	.09
Yes	328 (98.2)	6 (2.7)	0	
Free-standing ED or outpatient center				
No	79 (23.7)	50 (63.3)	29 (36.7)	.34
Yes	255 (76.3)	176 (69.0)	79 (31.0)	
Major teaching hospital				
No	261 (78.1)	167 (64.0)	94 (36.0)	.007
Yes	73 (21.9)	59 (80.8)	14 (19.2)	
Medical school affiliation				
No	122 (36.5)	73 (59.8)	49 (40.2)	.02
Yes	212 (63.5)	153 (72.2)	59 (27.8)	
Fee-for-service model				
No	309 (92.5)	211 (68.3)	98 (31.7)	.39
Yes	25 (7.5)	15 (60.0)	10 (40.0)	
Integrated salary model				
No	143 (42.8)	82 (57.3)	61 (42.7)	<.001
Yes	191 (57.2)	144 (75.4)	47 (24.6)	
Accountable care organization				
No	126 (42.6)	78 (61.9)	48 (38.1)	.03
Yes	170 (57.4)	125 (73.5)	45 (26.5)	
Total facility admissions				
Highest quintile	266 (79.9)	173 (65.0)	93 (35.0)	.05
Lowest 4 quintiles	67 (20.1)	52 (77.6)	15 (22.4)	
Total facility Medicaid discharges				
Highest quintile	266 (79.9)	176 (66.2)	90 (33.8)	.28
Lowest 4 quintiles	67 (20.1)	49 (73.1)	18 (26.9)	
Hospital overall star rating				
1, lowest	22 (6.6)	16 (72.7)	6 (27.3)	.32
2	76 (22.9)	49 (64.5)	27 (35.5)	
3	85 (25.6)	60 (70.6)	25 (29.4)	
4	98 (29.5)	71 (72.4)	27 (27.6)	
5, highest	51 (15.4)	29 (56.9)	22 (43.1)	
Effectiveness of care, national average				
Above	15 (4.5)	6 (40.0)	9 (60)	.03
Same	280 (84.6)	190 (67.9)	90 (32.1)	
Below	36 (10.9)	28 (77.8)	8 (22.2)	
Timeliness of care, national average				
Above	46 (13.9)	29 (63.0)	17 (37.0)	.68
Same	108 (32.6)	72 (66.7)	36 (33.3)	
Below	177 (53.5)	123 (69.5)	54 (30.5)	

Abbreviations: CoC, Commission on Cancer; ED, emergency department; NCI, National Cancer Institute.

### Facility-Level Characteristics and Factors Associated With Medicaid Acceptance by Cancer Site

We further evaluated characteristics associated with Medicaid acceptance by cancer site. Hospital characteristics found to be significant on univariable analysis are shown in Table 3. Several facility-level factors were associated with Medicaid acceptance across multiple cancer types, including state expansion status, facility, type, ownership, and ACO status. Multivariable analysis demonstrated similar findings (Table 4). State expansion status was associated with Medicaid acceptance for breast and kidney cancer care. Comprehensive community cancer programs were significantly less likely to accept Medicaid than community cancer programs for colorectal, kidney, and breast cancer. An integrated salary model was associated with Medicaid acceptance for colorectal and skin cancer.

**Table 3.** Univariable Analysis of Hospital Characteristics Associated With Medicaid Acceptance for Colorectal, Breast, Kidney, and Skin Cancer

Hospital characteristics	Colorectal		Breast		Kidney		Skin (melanoma)	
	Facilities, No./total No. (%)	*P* value	Facilities, No./total No. (%)	*P* value	Facilities, No./total No. (%)	*P* value	Facilities, No./total No. (%)	*P* value
State expansion status								
Nonexpansion state	89/104 (85.6)	.04	92/104 (88.5)	<.001	81/104 (77.9)	.003	78/104 (75.0)	.13
Expansion state	213/230 (92.6)		227/230 (98.7)		209/230 (90.9)		188/230 (81.7)	
Facility type								
Community	71/75 (94.7)	.03	72/75 (96)	.76	69/75 (92)	.02	64/75 (85.3)	<.001
NCI designated	27/29 (93.1)		28/29 (96.6)		27/29 (93.1)		29/29 (100)	
Integrated network	34/36 (94.4)		33/36 (91.7)		32/36 (88.9)		28/36 (77.8)	
Academic comprehensive	43/44 (97.7)		43/44 (97.7)		42/44 (95.5)		40/44 (90.9)	
Comprehensive community	127/150 (84.7)		143/150 (95.3)		120/150 (80.0)		105/150 (70.0)	
Ownership								
For-profit	30/39 (79.0)	.04	34/39 (89.5)	.08^a^	26/39 (68.4)	<.001	22/39 (57.9)	<.001
Non-government non-profit	236/257 (91.8)		246/257 (95.7)		226/257 (87.9)		209/257 (81.3)	
Government	36/39 (92.3)		39/39 (100.0)		38/39 (97.4)		35/39 (89.7)	
Sole community provider								
No	6/6 (100.0)	.42	6/6 (100.0)	.59	6/6 (100.0)	.34	6/6 (100.0)	.21
Yes	296/328 (90.2)		313/328 (95.4)		284/328 (86.6)		260/328 (79.5)	
Free-standing ED or outpatient center								
No	69/79 (87.3)	.29	77/79 (97.5)	.34	70/79 (88.6)	.59	57/79 (72.2)	.05
Yes	233/255 (91.4)		242/255 (94.9)		220/255 (86.3)		209/255 (82.0)	
Major teaching hospital								
No	234/261 (89.7)	.37	250/261 (95.8)	.64	221/261 (84.7)	.03	198/261 (75.9)	<.001
Yes	68/73 (93.2)		69/73 (94.5)		69/73 (94.5)		68/73 (93.2)	
Medical school affiliation								
No	109/122 (89.3)	.61	115/122 (94.3)	.41	99/122 (81.1)	.02	95/122 (77.9)	.49
Yes	193/212 (91.0)		204/212 (96.2)		191/212 (90.1)		171/212 (80.7)	
Fee-for-service model								
No	278/309 (90.0)	.32	296/309 (95.8)	.38	268/309 (86.7)	.86	247/309 (79.9)	.61
Yes	24/25 (96.0)		23/25 (92.0)		22/25 (88.0)		19/25 (76.0)	
Integrated salary model								
No	121/143 (84.6)	.002	136/143 (95.1)	.76	122/143 (85.3)	.48	102/143 (71.3)	.002
Yes	181/191 (94.8)		183/191 (95.8)		168/191 (88.0)		164/191 (85.9)	
Accountable care organization								
No	111/126 (88.1)	.07^a^	117/126 (92.9)	.05	107/126 (84.9)	.40	95/126 (75.4)	.03
Yes	160/170 (94.1)		166/170 (97.6)		150/170 (88.2)		144/170 (84.7)	
Total facility admissions								
Highest quintile	241/266 (90.6)	.79	254/266 (95.5)	.99	227/266 (85.3)	.12	207/266 (77.8)	.07^a^
Lowest 4 quintiles	60/67 (89.6)		64/67 (95.5)		62/67 (92.5)		58/67 (86.6)	
Total facility Medicaid discharges								
Highest quintile	241/266 (90.6)	.79	256/266 (96.2)	.19	228/266 (85.7)	.25	210/266 (78.9)	.43
Lowest 4 quintiles	60/67 (89.6)		62/67 (92.5)		61/67 (91.0)		55/67 (82.1)	
Hospital overall star rating								
1, lowest	19/22 (86.4)	.39	20/22 (90.9)	.02	21/22 (95.5)	.35	17/22 (77.3)	.07^a^
2	69/76 (90.8)		70/76 (92.1)		64/76 (84.2)		60/76 (78.9)	
3	75/85 (88.2)		81/85 (95.3)		74/85 (87.1)		74/85 (87.1)	
4	93/98 (94.9)		98/98 (100.0)		88/98 (89.8)		80/98 (81.6)	
5, highest	44/51 (86.3)		48/51 (94.1)		41/51 (80.4)		34/51 (66.7)	
Effectiveness of care, national average								
Above	13/15 (86.7)	.86	15/15 (100.0)	.57	7/15 (46.7)	<.001	9/15 (60.0)	.03
Same	253/280 (90.4)		266/280 (95.0)		248/280 (88.6)		222/280 (79.3)	
Below	33/36 (91.7)		35/36 (97.2)		32/36 (88.9)		33/36 (91.7)	
Timeliness of care, national average								
Above	43/46 (93.5)	.68	44/46 (95.7)	.99	38/46 (82.6)	.47	34/46 (73.9)	.31
Same	96/108 (88.9)		103/108 (95.4)		92/108 (85.2)		84/108 (77.8)	
Below	160/177 (90.4)		169/177 (95.5)		157/177 (88.7)		146/177 (82.5)	

Abbreviations: ED, emergency department; NCI, National Cancer Institute.

^a^ Approaching significance on univariable analysis (*P* < .10).

**Table 4.** Multivariate Logistic Regression of Key Hospital Characteristics Associated With Medicaid Acceptance^a^

Hospital characteristics	Colorectal		Breast		Kidney		Skin (melanoma)		All 4 cancer types (high access)	
	OR (95% CI)	*P* value	OR (95% CI)	*P* value	OR (95% CI)	*P* value	OR (95% CI)	*P* value	OR (95% CI)	*P* value
State expansion status										
Nonexpansion (n = 104)	1 [Reference]	NA	1 [Reference]	NA	1 [Reference]	NA	NA^b^	NA^b^	1 [Reference]	NA
Expansion (n = 230)	1.2 (0.5-3.1)	.69	15.6 (3.2-76.9)	<.001	2.3 (1.1-4.9)	.03	NA^b^	NA^b^	1.3 (0.7-2.3)	.47
Facility type										
Community (n = 75)	1 [Reference]	NA	NA^b^	NA^b^	1 [Reference]	NA	1 [Reference]	NA	1 [Reference]	NA
NCI designated (n = 29)	0.3 (0.0-2.3)	.23	NA^b^	NA^b^	0.3 (0.0-3.4)	.35	NA^c^	NA^c^	3.4 (0.6-19.0)	.16
Integrated network (n = 36)	0.4 (0.0-3.1)	.37	NA^b^	NA^b^	0.5 (0.1-2.2)	.35	0.7 (0.2-2.6)	.60	0.8 (0.3-2.3)	.63
Academic comprehensive (n = 44)	0.9 (0.1-11.3)	.95	NA^b^	NA^b^	0.7 (0.1-5.2)	.69	1.3 (0.2-7.4)	.78	3.2 (0.7-13.7)	.12
Comprehensive community (n = 150)	0.2 (0.0-0.9)	.04	NA^b^	NA^b^	0.2 (0.1-0.6)	.006	0.4 (0.2-0.9)	.04	0.4 (0.2-0.7)	.007
Ownership										
For-profit (n = 38)	1 [Reference]	NA	1 [Reference]	NA	1 [Reference]	NA	1 [Reference]	NA	1 [Reference]	NA
Nongovernment nonprofit (n = 257)	1.4 (0.3-5.9)	.67	1.2 (0.2-6.1)	.80	1.8 (0.7-5.2)	.66	3.0 (1.0-9.3)	.05	3.5 (1.1-10.8)	.03
Government (n = 39)	1.8 (0.3-11.2)	.54	NA^c^	NA^c^	8.8 (1.0-79.9)	.96	8.8 (1.7-46.1)	.01	6.6 (1.6-27.2)	.01
Integrated salary model										
No (n = 143)	1 [Reference]	NA	NA^b^	NA^b^	1 [Reference]	NA	1 [Reference]	NA	1 [Reference]	NA
Yes (n = 191)	2.8 (1.2-6.7)	.02	NA^b^	NA^b^	1.0 (0.5-2.2)	.91	2.3 (1.2-4.5)	.01	2.6 (1.5-4.5)	.001
Hospital overall star rating										
1, lowest (n = 22)	NA^b^	NA^b^	1 [Reference]	NA	NA^b^	NA^b^	1 [Reference]	NA	NA^b^	NA^b^
2 (n = 76)	NA^b^	NA^b^	0.6 (0.1-6.9)	.67	NA^b^	NA^b^	4.6 (1.0-22.0)	.06	NA^b^	NA^b^
3 (n = 85)	NA^b^	NA^b^	1.1 (0.1-12.7)	.94	NA^b^	NA^b^	5.5 (1.2-26.1)	.03	NA^b^	NA^b^
4 (n = 98)	NA^b^	NA^b^	NA^c^	NA^c^	NA^b^	NA^b^	3.8 (0.8-17.4)	.08	NA^b^	NA^b^
5, highest (n = 51)	NA^b^	NA^b^	0.6 (0.0-7.9)	.70	NA^b^	NA^b^	1.7 (0.4-8.0)	.51	NA^b^	NA^b^
Effectiveness of care, national average										
Above (n = 15)	NA^b^	NA^b^	NA^b^	NA^b^	1 [Reference]	NA	1 [Reference]	NA	1 [Reference]	NA
Same (n = 280)	NA^b^	NA^b^	NA^b^	NA^b^	8.7 (2.3-32.9)	.001	2.0 (0.4-11.4)	.41	6.4 (1.4-29.6)	.02
Below (n = 36)	NA^b^	NA^b^	NA^b^	NA^b^	7.6 (1.4-41.4)	.02	4.4 (0.5-37.5)	.18	8.4 (1.5-47.5)	.02

Abbreviations: NA, not applicable; NCI, National Cancer Institute; OR, odds ratio.

^a^ Only variables found to be significant on multivariable analysis are included in the table above. The following variables were also analyzed in the multivariable model: accountable care organization for colorectal cancer; accountable care organization for breast cancer; major teaching hospital and medical school affiliation for kidney cancer; freestanding emergency department or outpatient center, major teaching hospital, accountable care organization, and total facility admissions for skin cancer; and major teaching hospital, medical school affiliation, accountable care organization, and total facility admissions for high-access hospitals.

^b^ Association did not approach significance (*P* < .10) on univariable analysis and was not included in the multivariable model.

^c^ Variables where values demonstrated 100% Medicaid acceptance did not produce meaningful ORs on logistic regression.

### Facility-Level Factors Associated With High Medicaid Acceptance

In multivariable analysis shown in Table 4, comprehensive community cancer programs (OR, 0.4; 95% CI, 0.2-0.7; *P* = .002) had lower odds of offering high access to Medicaid compared with community cancer programs. Nongovernment, nonprofit (OR, 3.5; 95% CI, 1.1-10.8; *P* = .03) and government (OR, 6.6; 95% CI, 1.6-27.2; *P* = .01) facilities had greater odds of being high access compared with for-profit facilities. An integrated salary model (OR, 2.6; 95% CI, 1.5-4.5; *P* = .001) was associated with high access for patients with Medicaid. Hospitals with average (OR, 6.4; 95% CI, 1.4-29.6; *P* = .02) and below-average (OR, 8.4; 95% CI, 1.5-47.5; *P* = .02) effectiveness of care also had higher Medicaid access.

## Discussion

We conducted a national secret shopper study to evaluate access to new cancer care services for patients with Medicaid. We found that Medicaid acceptance differed widely across facilities, underscoring geographic variations in access for newly diagnosed patients with cancer seeking care at centers designated for cancer specialization. Medicaid acceptance practices also varied considerably within institutions, with only 67.7% of facilities providing access for Medicaid patients for all 4 cancer types examined. Differences in Medicaid acceptance based on specialty within institutions suggest opportunities for standardization to improve local consistency in access. Facility characteristics were also associated with Medicaid access, with the lowest acceptance at comprehensive community cancer centers, for-profit hospitals, and hospitals with above-average effectiveness of care and increased access among those with integrated salary models. Collectively, these findings underscore persistent gaps that exist for patients with Medicaid in accessing hospitals distinguished for high-quality cancer care.

Although almost all hospitals provided access for at least 1 cancer type, only 67.7% of hospitals surveyed offered care for all investigated cancer types. These findings may suggest that acceptance policies are primarily driven by individual departments rather than a singular facility standard, indicating that practice preference may play a role in Medicaid acceptance.^24^ In the era of multidisciplinary approaches to cancer treatment, variation in Medicaid acceptance within an institution may lead to increasingly fragmented care, which has been associated with lower quality care for several cancer types including rectal cancer.^25,26^ These findings indicate that greater consistency in Medicaid acceptance within institutions can also be viewed as a component of higher-quality, integrated care.

Findings of incomplete and highly variable Medicaid acceptance across CoC-accredited hospitals provides an important context through which to interpret the effects of ACA-related Medicaid expansions. To date, key estimates of ACA-related effects have been drawn from the National Cancer Database, a hospital registry source aggregating data from CoC-accredited hospitals and capturing approximately 70% of cancer care delivered in the United States.^8,27,28,29^ From this perspective, our study, focused at the level of patient access, reveals significant barriers to entry at CoC hospitals and indicates potential sources of representation bias by the systematic exclusion of patients with Medicaid at certain institutions. Insurance status overlaps heavily with other sources of sociodemographic disparities in cancer care. Therefore, subsequent investigation should evaluate the extent to which varied Medicaid acceptance could widen sociodemographic disparities in cancer care, such as those relating to race and ethnicity.^30^

We found that organizational and financial characteristics of facilities were drivers of Medicaid access. Comprehensive community cancer programs, or programs that assess 500 or more newly diagnosed cancer cases annually, were less likely to accept Medicaid.^15^ This finding is concordant with trends in literature, which show that patients with Medicaid may face significant financial and logistic barriers to care and are less likely to receive surgical care at high-volume centers.^31,32,33,34,35,36^ Given that patients with Medicaid typically have complex health needs, worse outcomes, and lower reimbursement rates, performance and revenue-conscious facilities with access to more favorably insured patients may continue to exercise greater selectivity and exclude patients with Medicaid.^24,33,37,38^ Reduced access to high-volume centers may, in turn, generate self-fulfilling cycles of adverse outcomes for patients with Medicaid.^39,40,41,42,43,44^ Greater complexity may also contribute to the below-average effectiveness of care of high-access facilities.^45^ Academic, NCI-designated, and integrated network cancer programs also had lower access for patients with Medicaid. Reduced access in these settings may serve to limit clinical trial participation and magnify racial and ethnic disparities in cancer care.^46,47,48,49^

Higher rates of Medicaid acceptance for breast and colorectal cancer may be the result of legislative actions supporting cancer screening services. A stipulation of the ACA requires insurers, including many Medicaid plans, to cover health care services recommended under the United States Preventative Services Task Force and the Health Resources and Services Administration guidelines without any patient cost-sharing, including breast and colorectal cancer screening.^50,51,52^ In addition, The Women’s Health and Cancer Rights Act of 1998 mandates coverage of interventions such as reconstructive surgery following breast cancer.^51^ While mandates do not require health system or hospital participation, they may facilitate broader coverage for these services. The benefits of current mandates suggest that expanding mandates for coverage of cancer care may prove effective and should be considered. Comparatively reduced access to skin and kidney cancer care is consistent with known barriers to surgical subspeciality care.^53^

Strategies to address persistent access barriers to cancer care for patients with Medicaid will require interventions at multiple levels. Lower reimbursement, often below the costs of delivering care, is a key barrier to Medicaid acceptance.^54^ With an increase in the number of patients with Medicaid, private systems may view even small changes in their payer mix as a serious financial risk, which may further restrict access in the coming years.^55^ Therefore, increases in reimbursement rates should continue to be prioritized, as even modest increases in Medicaid reimbursement rates have been shown to increase the likelihood of Medicaid acceptance.^56,57^ Indeed, increasing Medicaid reimbursement by as little as $10 per visit significantly increased the probability of a physician visit, and closing payment gaps between Medicaid and private insurance eliminated more than two-thirds of access disparities among adults.^56,57^ However, barriers to Medicaid acceptance extend beyond reimbursement rates alone, including administrative burden (eg, billing complications, delayed payments), complex patient needs, and capacity restraints.^4,11,56,57^ Thus, continued progress in payment reform models that incentivize health care quality are also a promising strategy to improve access.^58,59^

We observed that facilities with integrated salary models had greater access for patients with Medicaid, calling further attention to the importance of payment structures in Medicaid acceptance, as physicians within integrated systems may be more willing to see patients with Medicaid when compensation is based on salary or work-relative value units regardless of patient insurance status.^60^ Volume of patients covered by Medicaid managed care plans (total facility Medicaid discharges) was also not significantly associated with Medicaid acceptance in this study, suggesting room for improvement in financial incentive models for care for patients with Medicaid. New payment models for Medicaid, such as risk-based managed care or pay-for-performance payment models, are also promising strategies for improving access and outcomes for patients with cancer who are insured by Medicaid.

### Limitations

There are limitations to this study. Measuring access to care is practically limited because of the range in care pathways (eg, emergency department, referrals) and entry and exit points along care continuum (eg, initial appointment, surgery, pharmaceutical treatment, follow-up). As a result, we used acceptance of Medicaid for an initial appointment as a proxy to measure access to care. Additionally, we did not contact all CoC-accredited facilities; however, our random sample captured one-third of CoC-accredited facilities and provided a representative sampling to evaluate factors associated with access to care. The timing of this study also coincided with the COVID-19 pandemic, which may have impacted acceptance rates owing to lower service utilization and facility-level restrictions on approved health care services, respectively. This study also did not include a control group of simulated patients with Medicare or commercial insurance. Therefore, we are unable to draw direct comparisons to assess the differential effects of insurance status, although acceptance rates for patients with commercial insurance have been reported as high as 99% in the literature.^11,20,61^ Additionally, given that this study was limited to CoC-accredited facilities, we did not evaluate overall access to cancer services in the United States.

## Conclusions

In this national cross-sectional study, we found that disparities in access to cancer care for patients with Medicaid exist both within and across hospitals in the United States. This study uncovered facility-level variability in access to cancer care for patients with Medicaid. Furthermore, we found that access was associated with facility-level factors, including facility type, facility ownership, integrated salary model, and effectiveness of care. Facility-level differences in Medicaid access among CoC facilities are notable given the use of hospital registry data and CoC-accredited hospital databases to estimate ACA-related outcomes. Given that patients with Medicaid may be preferentially denied access, future studies should account for potential sources of bias contributed by insurance status. To our knowledge, this is the first national cross-sectional study to comprehensively investigate Medicaid acceptance and specific state and facility-level factors that may affect Medicaid acceptance across cancer types at cancer hospitals. Further investigation is warranted to clarify and address access barriers to cancer care for patients with Medicaid.

## Notes

Supplement. **eFigure.** Flowchart of Facility Sample
