# Supplementary material for: Acceptance of Simulated Adult Patients With Medicaid Insurance Seeking Care in a Cancer Hospital for a New Cancer Diagnosis
Source: JAMA Netw Open. 2022 Jul 15;5(7):e2222214. doi: 10.1001/jamanetworkopen.2022.22214 (PMC9287756; doi:10.1001/jamanetworkopen.2022.22214)
Supplement: Supplement. — eFigure. Flowchart of Facility Sample [file jamanetwopen-e2222214-s001.pdf]

Supplemental Online Content

Marks VA, Hsiang WR, Nie J, et al. Acceptance of simulated adult patients with Medicaid insurance seeking care in a cancer hospital for a new cancer diagnosis. *JAMA Netw Open.* 2022;5(7):e2222214. doi:10.1001/jamanetworkopen.2022.22214

eFigure. Flowchart of Facility Sample

This supplemental material has been provided by the authors to give readers additional information about their work.

© 2022 Marks VA et al. *JAMA Network Open.*

eFigure. Flowchart of Facility Sample

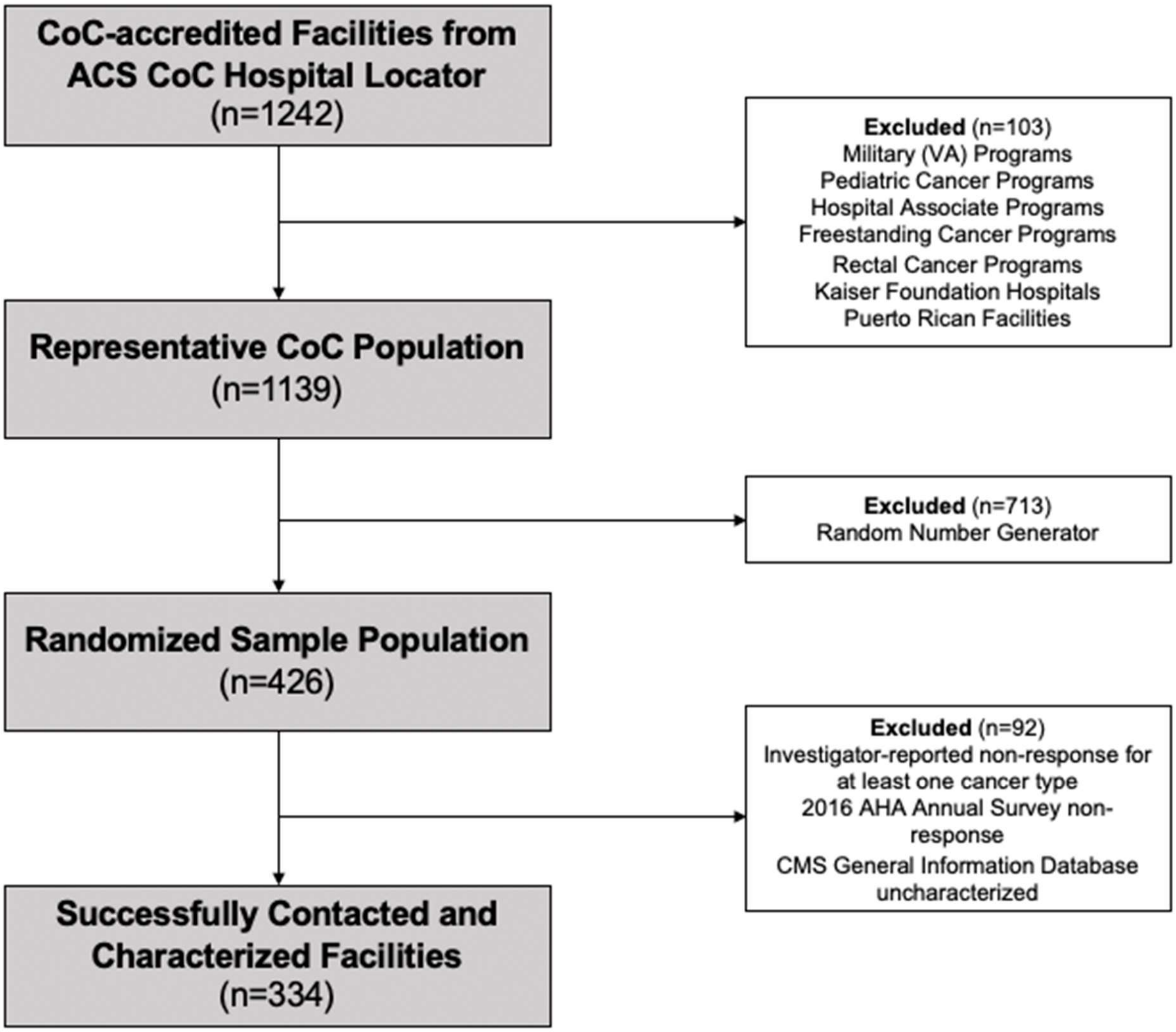

ACS: American College of Surgeons; AHA: American Hospital Association; CMS: Centers for Medicare & Medicaid Services; CoC: Commission on Cancer; VA: Veterans Affairs.

© 2022 Marks VA et al. *JAMA Network Open.*
